# Formation and Transformation Behavior of Sodium Dehydroacetate Hydrates

**DOI:** 10.3390/molecules21040458

**Published:** 2016-04-06

**Authors:** Xia Zhang, Chuang Xie, Yaohui Huang, Baohong Hou, Ying Bao, Junbo Gong, Qiuxiang Yin, Sohrab Rohani

**Affiliations:** 1 State Key Laboratory of Chemical Engineering, School of Chemical Engineering and Technology, Tianjin University, No. 92 Weijin Road, Nankai District, Tianjin 300072, China; zhangxia@tju.edu.cn (X.Z.); acxie@tju.edu.cn (C.X.); hyhyezi@163.com (Y.H.); houbaohong@tju.edu.cn (B.H.); yingbao@tju.edu.cn (Y.B.); junbo_gong@tju.edu.cn (J.G.); 2 Collaborative Innovation Center of Chemical Science and Chemical Engineering (Tianjin), Tianjin 300072, China; 3 Department of Chemical and Biochemical Engineering, The University of Western Ontario, London, ON N6A 5B9, Canada

**Keywords:** sodium dehydroacetate, monohydrate, dihydrate, cooling crystallization, hollow crystal, formation mechanism, transformation

## Abstract

The effect of various controlling factors on the polymorphic outcome of sodium dehydroacetate crystallization was investigated in this study. Cooling crystallization experiments of sodium dehydroacetate in water were conducted at different concentrations. The results revealed that the rate of supersaturation generation played a key role in the formation of the hydrates. At a high supersaturation generation rate, a new sodium dehydroacetate dihydrate needle form was obtained; on the contrary, a sodium dehydroacetate plate monohydrate was formed at a low supersaturation generation rate. Furthermore, the characterization and transformation behavior of these two hydrated forms were investigated with the combined use of microscopy, powder X-ray diffraction (PXRD), Raman spectroscopy, Fourier transform infrared (FTIR), thermal gravimetric analysis (TGA), scanning electron microscopy (SEM) and dynamic vapor sorption (DVS). It was found that the new needle crystals were dihydrated and hollow, and they eventually transformed into sodium dehydroacetate monohydrate. In addition, the mechanism of formation of sodium dehydroacetate hydrates was discussed, and a process growth model of hollow crystals in cooling crystallization was proposed.

## 1. Introduction

The formation of polymorphs, solvates and hydrates is a prevalent phenomenon in the production of chemical compounds including active pharmaceutical ingredients [1,2]. Different polymorphs of a chemical compound may exhibit different physicochemical properties such as solubility, melting point and density. Like polymorphs, solvates and hydrates may also impart different chemical and physical properties. Production of an unwanted hydrate will not satisfy the intended purpose or processing characteristics of the final product [2,3]. Therefore, it is of utmost importance to identify and understand the conditions necessary to form a particular hydrate as well as the transformation pathways to other solid phases such as other hydrates and anhydrous phases [4,5].

In recent years, the use of seeding, structurally similar additives and different solvents have been widely adopted to induce polymorph formation. Besides the abovementioned factors, the rate of supersaturation generation usually plays a significant role in determining the polymorphic outcome [6], especially in cooling crystallization. He *et al.* [7] described that kinetic effects were dominant when the rate of supersaturation generation was high, thereby producing the metastable form II of *o*-aminobenzoic acid; while the more stable forms III and I of *o*-aminobenzoic acid were formed when the rate of supersaturation generation was low. Barrett *et al.* [8] also observed that the form II polymorph of piracetam was produced at fast cooling rates, and the stable form III polymorph was produced with a slow cooling rate. However, to the best of our knowledge, there is no reports on the rate of supersaturation generation on the crystallization of hydrates.

In the literature, hollow crystals, also called hollow needles, hollow whiskers, tubular crystals, *etc.*, result from particular crystal growth conditions [9]. High porosity and specific surface area are attractive for applications in catalysis and drug delivery [10]. Dimethyl sulfoxide or dimethylformamide solvates of dexamethasone acetate [11] and sodium-2-keto-L-gulonate hydrate crystals [12] have been reported in the literature to form hollow crystalline needles during a solvent-mediated phase transformation. The anti-solvent method and evaporative crystallization can also be used to obtain hollow crystals of molecules such as deflazacort (DFZ) and carbamazepine (CBZ) [13,14]. However, no account on the use of cooling methods to prepare hollow crystals has been reported in the literature.

**Figure 1:**
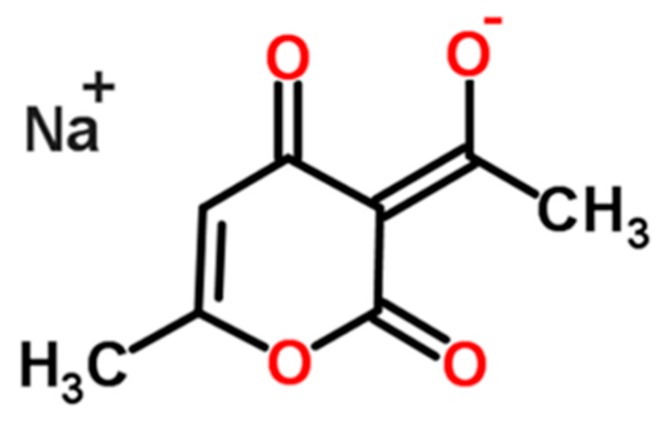
Molecular structure of sodium dehydroacetate.

According to a survey of the Cambridge Structural Database (CSD) Version 5.26 by Motherwell and coworkers [3], approximately 6.5% (6558) of the crystal structures in the database (101,244) are in a hydrated form [3]. In this paper, the work has been focused on the crystallization of a sodium salt, sodium dehydroacetate (DHA-S). The molecular structure of DHA-S is shown in Figure 1. This compound is a new type of food preservative, which is safe and has a strong antimicrobial activity against foodborne pathogens and spoilage organisms. In our earlier reports [5], DHA-S was shown to be capable of existing in anhydrous and monohydrate solid state forms, and phase transformation between these two forms was studied. The monohydrate of DHA-S is the commercial chemical compound because it is easy to produce using cooling crystallization. However, during the cooling crystallization experiments of DHA-S in water, a new needle dihydrate form of DHA-S was obtained by our team. In this work, the crystallization conditions of these two hydrates will be presented for the first time, and a comprehensive solid-state characterization of the dihydrate will be reported. Furthermore, the transformation behaviour of the monohydrate and dihydrate of DHA-S will be investigated. In addition, the influential variables and the formation mechanisms of different hydrates will be discussed. This is essential for crystallization process design in the chemical and pharmaceutical industries and isolation of desired polymorphs.

## 2. Results and Discussion

### 2.1. The Effect of Cooling Rate on Hydrate Forms

**Figure 2:**
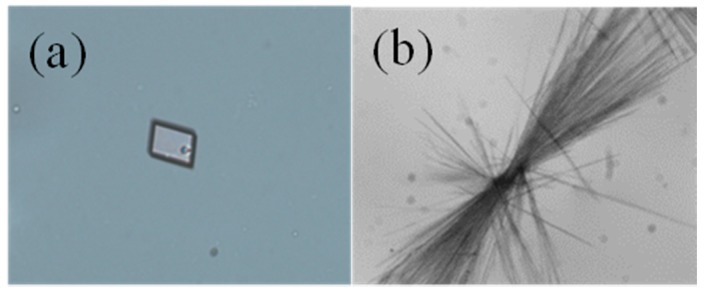
Optical micrographs taken at different cooling rates during the crystallization process: (**a**) 0.15 K/min; (**b**) 0.25 K/min.

The crystals resulting from controlled cooling crystallization of DHA-S/water solutions at different cooling rates were characterized by microscope, PXRD, Raman, FTIR and TGA. As shown in Figure 2, the crystal habit is plate-like when the cooling rate is 0.15 K/min, and the crystal habit becomes needle-like at 0.25 K/min cooling rate. In addition, the collected crystals contain both plate-like and needle-like when the cooling rate is 0.20 K/min. The plate-like crystal habit is similar to the habit of commercial DHA-S monohydrate.

**Figure 3:**
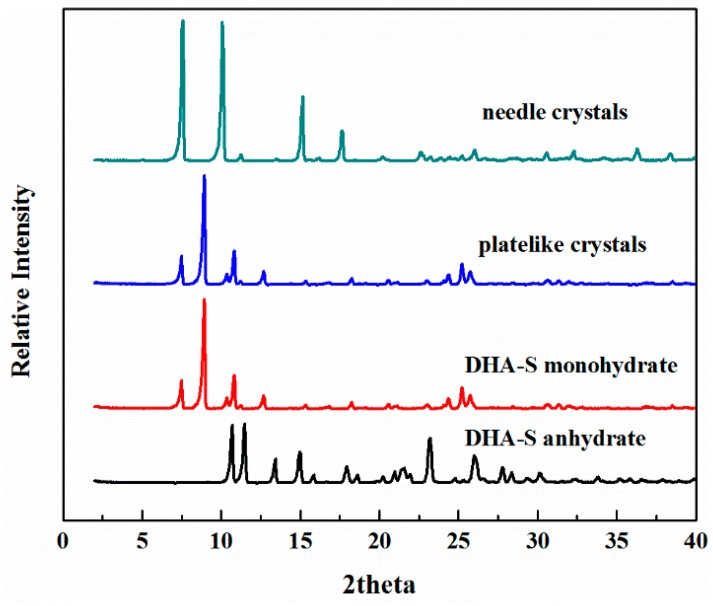
Powder X-ray diffraction patterns of DHA-S.

To compare the obtained crystals, the powder X-ray diffraction patterns of the anhydrous [5], commercial monohydrate and the crystals collected under different cooling rates were measured and are shown in Figure 3. The characteristic peak positions present in the PXRD for plate-like crystals are the same as those of the commercial monohydrate of DHA-S. That confirms that the DHA-S monohydrate crystals are obtained when the cooling rate is 0.15 K/min. In contrast, the PXRD characteristic peak positions of needle-shaped crystals are obviously different from the anhydrous and commercial monohydrate, confirming that these compounds are distinct.

**Figure 4:**
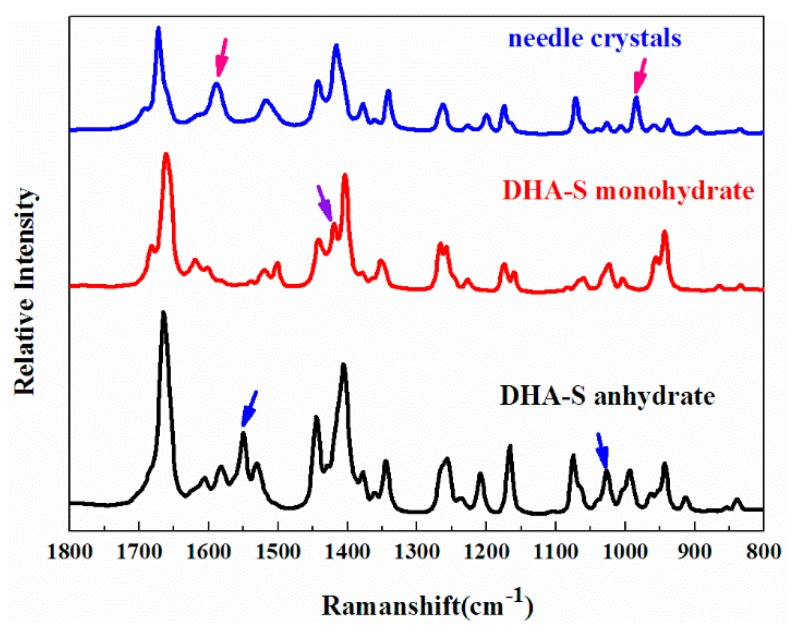
Raman spectra of DHA-S obtained from powders.

**Figure 5:**
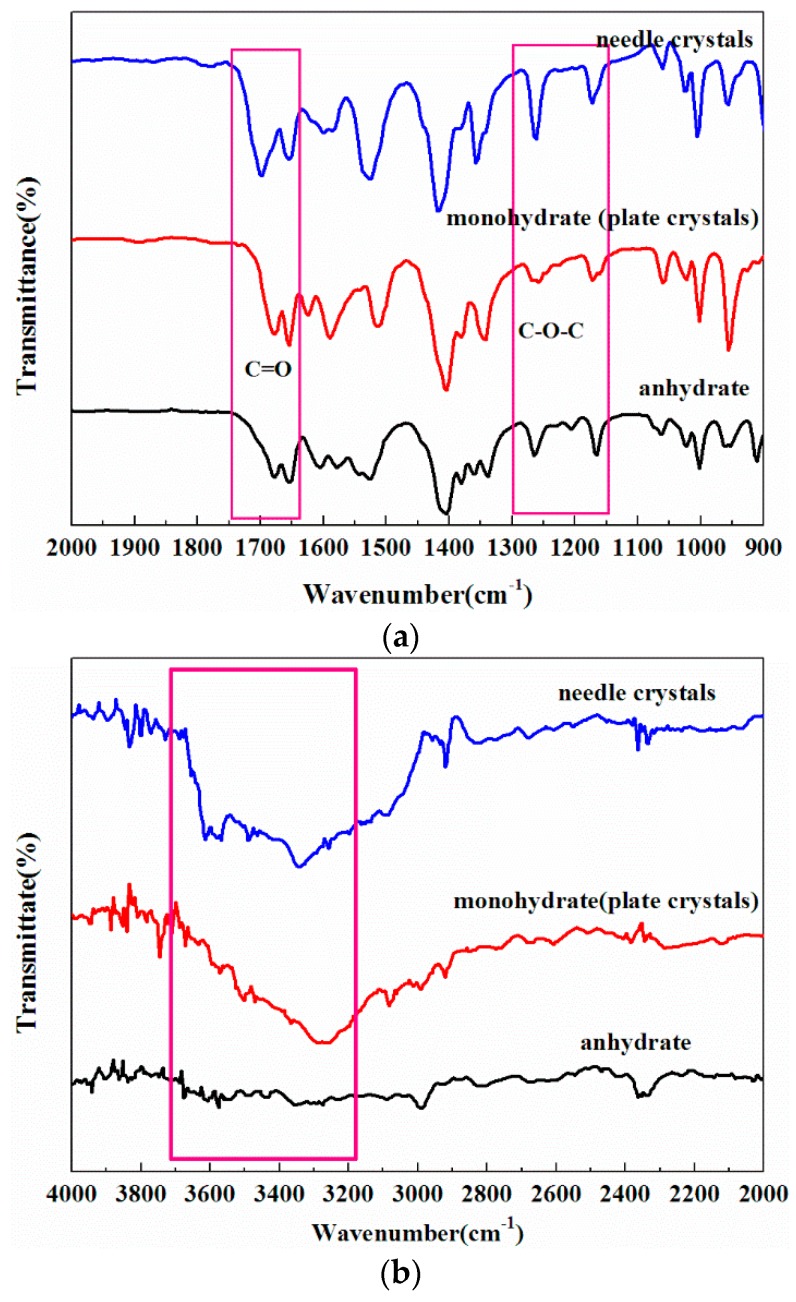
FTIR spectra of DHA-S: (**a**) spectra from 2000 to 900 cm^−1^; (**b**) spectra from 4000 to 2000 cm^−1^.

Raman spectroscopy was successfully applied to identify the obtained solid state products, as shown in Figure 4. The three forms show many different characteristic Raman peaks, as marked in the graph. In the mid-infrared spectrum (Figure 5) of different DHA-S solid forms, all three spectra have strong absorption bands at 1750–1640 cm^−1^ assigned to the carbonyl stretching vibration [15]. For the ester C=O stretching vibration, the stretching vibration occurs at 1750–1670 cm^−1^; whereas the isolated C=O is observed at 1670–1640 cm^−1^. At the same time, the two strong absorption bands of all the solid forms which occur in the range 1300–1100 cm^−1^ are due to the ester C-O-C stretching vibrations. By comparison, the major difference in the three spectra was the absorption region from 3700 to 3200 cm^−1^ assigned to the hydroxyl group of water in the crystals [15]. There is no absorption peak in DHA-S anhydrate, whereas the absorption band is very strong for DHA-S monohydrate and needle-like crystals. It is speculated that the needle crystals may also be a hydrate form.

**Figure 6:**
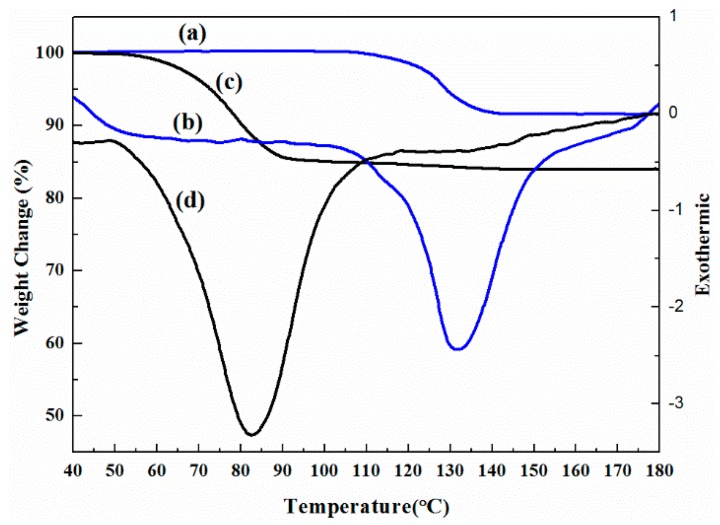
TGA and DSC curves of DHA-S: (**a**) TGA curve of monohydrate; (**b**) DSC curve of monohydrate; (**c**) TGA curve of needle crystals; (**d**) DSC curve of needle crystals.

The DSC and TGA curves of the monohydrate and needle-shaped crystals of sodium dehydroacetate are shown in Figure 6. The TGA thermograph of DHA-S monohydrate shows an 8.66% weight loss at around 120 °C [5]. In contrast, the endothermic peak and mass loss can be observed from the DSC and TG data of needle shape crystals at about 80 °C. The TGA thermograph indicates 15.61% weight loss of the needle crystals, and the mass loss is consistent with the calculated water content (15.93%) in DHA-S dihydrate.

The needle crystals can thus be identified as a DHA-S dihydrate according to the characterization, and the results of hydrate forms produced under different cooling rates are summarized in Table 1.

From the table, the hydrate outcome of the cooling crystallization of DHA-S in water has a close relationship with the cooling rate. The monohydrate crystals are obtained when the cooling rate is 0.15 K/min, and as the cooling rate is increased from 0.20 to 0.25 K/min, the dihydrate of DHA-S shows an increasing probability of formation. Knowledge of the crystallization conditions is essential when designing a process to isolate a desired hydrate form of DHA-S.

### 2.2. Phase Transformation Behaviour of DHA-S Hydrates

**Figure 7:**
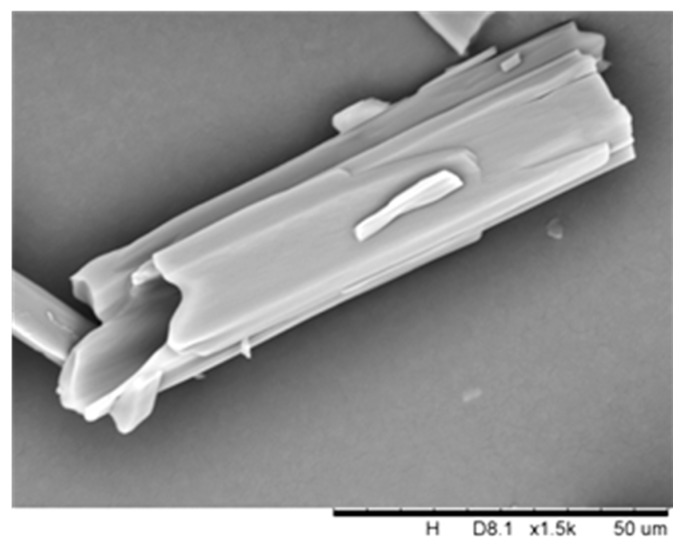
SEM image of DHA-S dehydrate.

More striking, the SEM images of DHA-S dihydrate indicate that the needle crystals are hollow and possess a smooth surface (Figure 7). The specific surface area of the DHA-S monohydrate and hollow dihydrate measured by their nitrogen adsorption isotherms are 15.31 m^2^/g and 21.37 m^2^/g, respectively. Indeed, the hollow dihydrate crystals generally exhibit a larger surface area than non-hollow crystals of DHA-S monohydrate. It is expected that the hollow dihydrate crystals may have better properties such as rapid dissolution rate compared to the monohydrate crystals. However, these hydrate crystals may undergo certain physical and chemical changes such as hydration–dehydration during environmental conditions changes and industrial processes [16]. In order to stabilize the DHA-S product quality, it is essential to investigate the possible transformation behaviours of DHA-S hydrates under different conditions.

**Figure 8:**
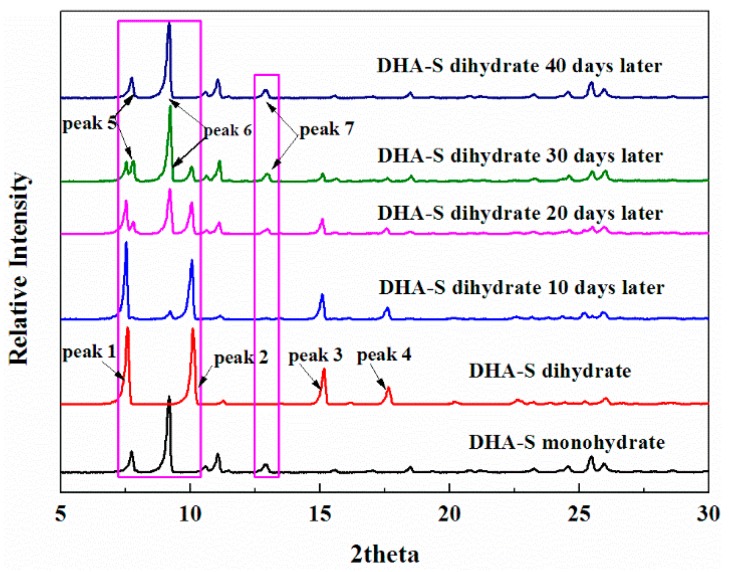
The changing powder X-ray diffraction patterns of DHA-S dihydrate upon exposure to 30% relative humidity at 25 °C for different time periods.

**Figure 9:**
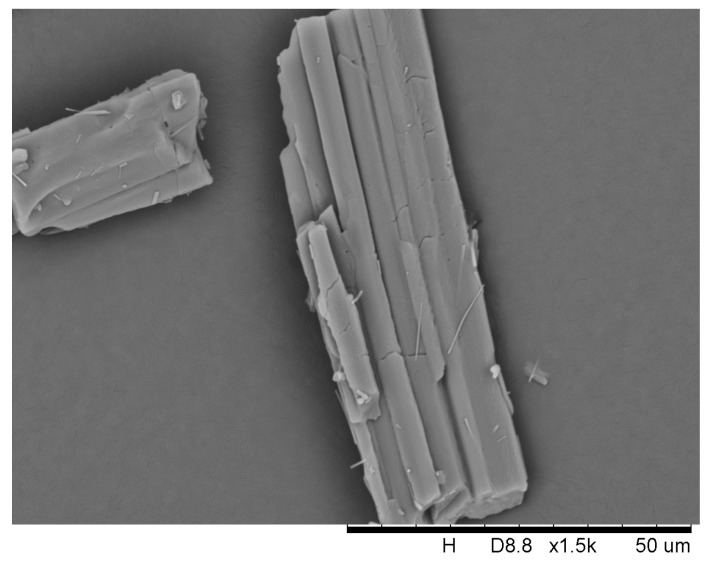
SEM image of DHA-S dihydrate after transformation into monohydrate.

The changes in the powder X-ray diffraction pattern intensity of the characteristic peaks of DHA-S dihydrate are shown in Figure 8. Maintaining the dihydrate at constant temperature and humidity in an incubator (25 °C with 30% relative humidity) for ten days, caused a decrease in the intensity of dihydrate’s main characteristic peaks (peaks 1–4). The intensity of these peaks gradually declined and they disappeared during the next one month. Meanwhile, the intensity of the monohydrate’s characteristic peaks (peaks 5–7) gradually increased. The decrease of the PXRD characterization peaks intensity of dihydrate and the increase of the PXRD characteristic peaks intensity of monohydrate phase suggest that the dihydrate transforms into monohydrate crystals. The interesting thing is that the crystal shape also changes after the transformation (Figure 9). The initial hollow shape of the dihydrate disappears and some cracks develop on the crystal surface. This can be explained by the removal of water molecules and molecular rearrangement in the crystal lattice leading to the changes of crystal shape [17,18,19].

**Figure 10:**
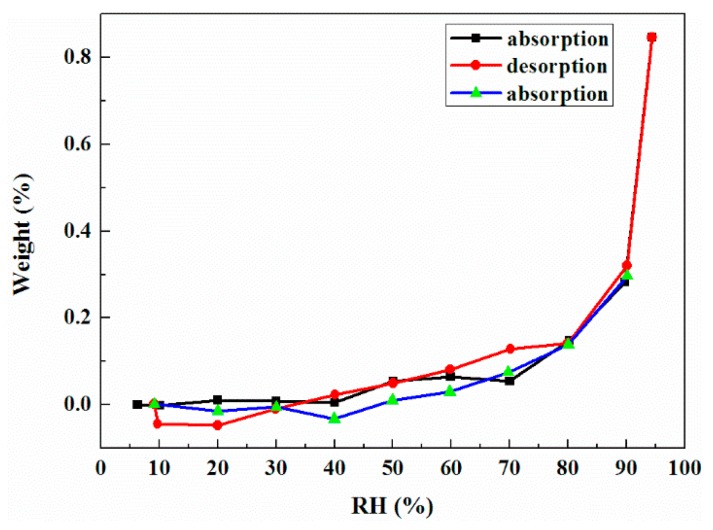
DVS plot of DHA-S monohydrate.

The isothermal hydration and dehydration behavior of DHA-S monohydrate is also displayed in Figure 10. DHA-S monohydrate shows a slight sorption of water over a considerable relative humidity range (5%–70%), and the mass is almost unchanged, suggesting that the monohydrate is a thermodynamically stable phase. When the relative humidity is over 70%, the change in mass of the sample remains below 1.0%. The adsorbed moisture is on the surface of monohydrate of DHA-S crsytals at high relative humidity.

Based on the crystallization behaviour and the lower dehydration temperature of dihydrate than monohydrate, it can be concluded that the DHA-S monohydrate is the thermodynamically stable form under the adopted experimental conditions.

### 2.3. Formation Mechanism of DHA-S Hydrates

The polymorphic outcome of the DHA-S hydrate forms is found to be cooling rate dependent. The monohydrate of DHA-S is more stable than the dihydrate. In general, fast cooling rates that correspond to higher rates of supersaturation generation have a greater tendency to form metastable polymorphs [20]. That’s why we get the more stable monohydrate form at slower cooling rates, and the less stable dihydrate at higher cooling rates. The nature of the solid-state phase is dependent on the driving force for crystallization, supersaturation. The level of supersaturation influences both the crystal nucleation and growth rates [20,21]. Generally, nucleation of the metastable form may be observed, even though it is not the thermodynamically favored phase. This is because nucleation of the metastable phase can occur at a faster rate and at higher levels of supersaturation generation.

In the cooling crystallization of DHA-S hydrates, temperature and supersaturation changed simultaneously, and the rate of supersaturation generation was adjusted by changing the rates of cooling. The cooling rate was higher for higher rate of supersaturation generation and *vice versa*. The dihydrate form crystallized first because the kinetic effects were dominant at the higher cooling rate, while the thermodynamic effects prevail in the crystallization of monohydrate crystals at the lower cooling rate. The metastable form of DHA-S dihydrate was subsequently transformed to the stable monohydrate provided that there was enough time to do so. This viewpoint is consistent with the experimental results.

**Figure 11:**
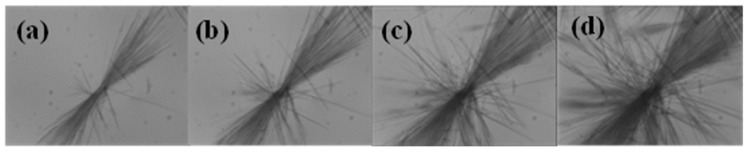
Optical micrographs taken at different time during the dihydrate crystals growth process: (**a**) 20 s; (**b**) 40 s; (**c**) 60 s; (**d**) 80 s.

**Figure 12:**
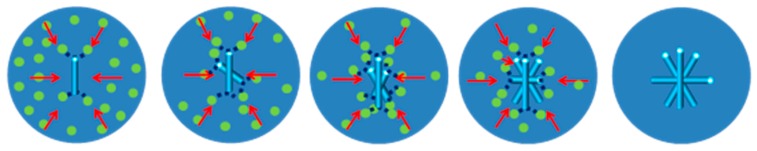
Proposed scheme of the growth mechanism of DHA-S hollow dehydrate.

The hollow crystal growth behaviour of DHA-S dihydrate was observed by optical microscopy (Figure 11). It was observed that hollow needle crystals preferentially appeared at the bottom, corners, edges, and defects of the container that generally are prone to higher heat dissipation rates. That is to say, these sites have a higher rates of supersaturation generation due to the higher rates of heat loss. On the other hand, the container defects constitute preferential sites for the nucleation and growth of hollow crystals. When the first hollow crystal appeared, a large number of filamentary particles irradiated from the initial particle. Then the crystals kept growing along one direction. Moreover, it is indicated that the dihydrate crystals growth rate in one direction is several times greater than that of the other directions at high supersaturation generation rates [22]. Eddleston and Jones [13] mentioned that two key factors of tubule formation are highly anisotropic crystal growth and high supersaturation levels in the evaporative crystallization. On the basis of these experimental observations, a growth mechanism of hollow crystals in cooling crystallization has been proposed. Figure 12 summarizes the main features of this mechanism, which consists of the following steps: The saturated solution of DHA-S/water at 343.15 K was pipetted into a 20 mL bottle and naturally-cooled at ambient temperature. Because of high supersaturation, dihydrate DHA-S crystallized immediately in the solution. Due to the high crystal growth rate of the dihydrate in one direction, this direction maintained a high growth rate when the saturated solution reached the surface of the initially appearing crystals. The diffusion and mass transport from the solution to the center of the growing crystal surface were inhibited and that led to the formation of a central cavity within the crystal. Meanwhile, some small crystals appear on the surface of the initial crystals with the molecular diffusion to the crystals surface. These crystals kept growing until the supersaturation level diminished.

## 3. Materials and Methods

### 3.1. Materials

Solid state sodium dehydroacetate monohydrate with mass fraction purity higher than 99% was purchased from Aladdin Chemistry Co. Ltd. (Shanghai, China), and was used without further purification. Deionized pure water prepared in our lab was used in all experiments.

### 3.2. Solid–State Characterization

Optical microscopy pictures were obtained by using a Eclipse E200 digital microscop eapparatus (Nikon, Tokyo, Japan) with a magnification of 40×. The PXRD spectra of the collected solid forms were obtained on a 2500 diffractometer (D/MAX, Rigaku, Japan) with a Cu Kα radiation source (λ = 1.5406 Å) at 100 mA and 40 kV. The samples were recorded at a scanning rate of 8° per minute over a 2θ range of 2–50°. The Raman spectra of the polymorphic transformation process was determined by a RAMAN RXN2 system (Kaiser Optical Systems, Inc., Ann Arbor, MI, USA). The Fourier transform infrared spectra were collected from KBr disks using a Nexus instrument (Thermo, Waltham, MA, USA), and ground KBr powder was used as the background in the measurements. The wavenumber range was measured from 4000 to 700 cm^−1^.

Thermal analysis was performed on a TGA/DSC1/SF (Mettler Toledo, Zurich, Switzerland). Approximately 5–10 mg of collected samples were heated from 298.15 K to 573.15 K at a rate of 10 K per minute with a dry nitrogen purging rate of 20 mL per minute. Scanning electron microscope (SEM) micrographs of collected solids were imaged by a TM3000 apparactus (Hitachi, Tokyo, Japan) at the voltage of 15 kV. The samples were gold sprayed with a thickness of 10 nm. The specific surface area was recorded via the Brunauer–Emmett–Teller (BET) method using a TriStar II 2020 porosimetry analyzer (Micromeritics, Norcross, GA, USA). The samples were degassed at 25 °C for 2 h prior to the measurements.

### 3.3. Controlled Cooling Crystallization Experiments

Suspensions of monohydrate DHA-S in water were separately prepared at 333.15 K and 343.15 K. The suspensions were kept at these temperatures for 5 hours to reach equilibrium. The suspensions were then filtered (0.2 mm filter) into a 100 mL jacketed crystallizer that was preheated to 333.15 K or 343.15 K, and a constant mechanical stirring speed (300 rpm) was used. The temperature of the solution was controlled using a thermostat bath. The solution was held at 333.15 K or 343.15 K for 30 min, then cooled to 293.15 K at different rates (0.15, 0.20 and 0.25 K/min). The resulting crystals were filtrated as soon as the crystallization processes were finished. All crystals were dried at 30 °C for about 4 h before characterization.

### 3.4. Solid–Solid Polymorphic Transformation of DHA-S Hydrates

The solid dihydrate of DHA-S was placed in a constant temperature and humidity incubator (25 °C with relative humidity 30%). Intermittent, certain samples were withdrawn for PXRD analysis every 10 days. SEM was used to observe the morphology of the monohydrate and dihydrate of DHA-S crystals.

The isotherm gravimetric water sorption and desorption processes of DHA-S monohydrate were carried out using a dynamic vapor sorption analyzer (VTI-SA, VTI Corporation, Irvine, CA, USA). The samples (20–30 mg) were placed in the DVS instrument at 25 °C, and the relative humidity (RH) was increased from 5% to 95% by steps of 10%, then decreased to 5% by steps of 10%.

### 3.5. Crystallization Behaviour of DHA-S Dihydrate

A saturation solution of DHA-S/water was prepared at 70 °C. Ten milliliters of the solution was filtered and pipetted into a 20 mL bottle kept at ambient temperature. The dihydrate crystal growth were monitored using optical microscopy by focusing on selected particles. The progressive evolution of crystals was collected by capturing images at 20 s intervals.

## 4. Conclusions

In this work, the effects of the rate of supersaturation generation on DHA-S hydrate crystallization were investigated through cooling crystallization experiments. The rate of supersaturation generation was altered by changing the cooling rate of DHA-S/water. The results revealed that the plate monohydrate of sodium dehydroacetate was formed at low generation rate of supersaturation, and a new needle dihydrate form of sodium dehydroacetate was obtained at a high supersaturation generation rate. This new form was characterized by using microscopy, PXRD, Raman, FTIR, TGA and SEM. It was found that the DHA-S dihydrate consists of hollow needle-shaped crystals. Furthermore, the transformation behavior between monohydrate and dihydrate was investigated. It can be concluded that the monohydrate is more stable than dihydrate. In addition, the experimental results suggest that the rate of supersaturation generation is the determining factor for the formation of different hydrates. A process growth model of hollow DHA-S dihydrate crystals in cooling crystallization was also proposed.

## Figures and Tables

**Table 1:** The results of hydrate forms under different cooling rates and initial saturation temperatures.

Cooling Rate	333.15 K Saturation Solution	343.15 K Saturation Solution
0.15 K/min	monohydrate	monohydrate
0.20 K/min	monohydrate and dihydrate	monohydrate and dihydrate
0.25 K/min	dihydrate	dihydrate
